# Achieving ultra-low oxygen transport resistance of fuel cells by microporous covalent organic framework ionomers

**DOI:** 10.1039/d5sc04070a

**Published:** 2025-10-06

**Authors:** Xiaoqin Ma, Xiaoli Lu, Shimei Liang, Caili Yuan, Jingtao Si, Jianchuan Wang, Zidong Wei

**Affiliations:** a School of Chemistry and Chemical Engineering, State Key Laboratory of Advanced Chemical Power Sources, Chongqing University 400044 Chongqing China jxw319@cqu.edu.cn; b Northwest Electric Power Design Institute Co., Ltd, of China Power Engineering Consulting Group Xi'an shaanxi Province 710075 China

## Abstract

Research on ultra-low platinum (Pt)-loaded fuel cells is essential for reducing costs and advancing hydrogen fuel cell commercialization. However, oxygen diffusion resistance remains a major challenge, limiting the oxygen reduction reaction and fuel cell efficiency. To address this challenge, a stable colloidal dispersion of polymer-grafted covalent organic framework (COF) ionomers has been developed. These COF ionomers enhance hydroxide conductivity and oxygen transport by creating a sub-nm porous structure on the catalyst surface, while also dispersing catalyst particles and stabilizing the three-phase interface. Compared to polymer electrodes, COF ionomer electrodes reduce oxygen transport resistance by 96.4%. With ultra-low Pt loading (60 μg cm^−2^), COF ionomer electrodes achieve a peak power density of 0.78 W cm^−2^, three times higher than that of polymer electrodes. This study presents a promising alternative for the development of more efficient ionomers with low oxygen transfer resistance in fuel cells.

## Abstract 2

COF ionomers form sub-nm porous structures on the catalyst surface, which shorten the oxygen diffusion path to the catalyst and enhance the utilization efficiency of platinum-based catalysts.

## Graphical abstract

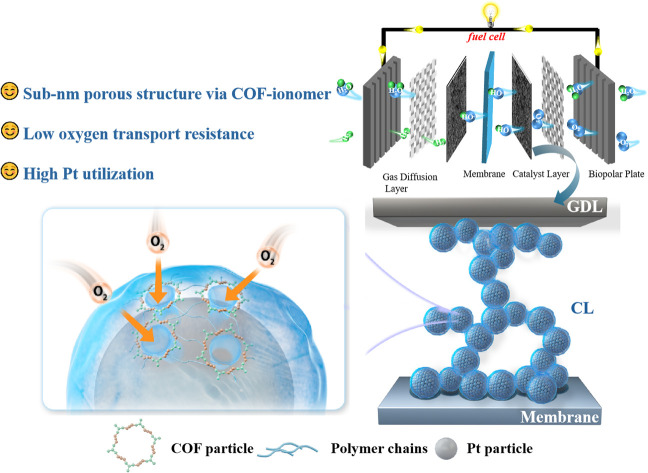

## Introduction

Hydrogen, a clean and efficient energy source, is the best choice to replace fossil fuels.^1,2^ Fuel cells, as highly efficient energy conversion devices for hydrogen energy, have attracted considerable attention and are very promising for commercialization.^3–5^ A great challenge is to reduce the cost of fuel cells to make them available as normal commodities.^6,7^ An expensive platinum group metal (PGM) catalyst is critical for energy conversion efficiency^8^ and in order to reduce the costs of fuel cells, reducing the load of PGM is imperative.^9–11^ As the Pt loading decreases, the effective surface area of the catalyst also decreases, leading to an increased demand for oxygen transport per catalyst.^12,13^ At this point, the local oxygen mass transfer resistance (*R*_local_), which is the resistance of oxygen though the ionomer thin film to the catalyst active sites, becomes the dominant factor influencing the oxygen reduction reaction (ORR) rate at ultra-low PGM loading.^14–16^

Therefore, designing ionomers that have low oxygen transfer resistance in the CL is essential.^17^ The traditional approach involved increasing groups with highly oxygen-dissolving capacity^18–20^ or modifying the phase separation to improve oxygen self-diffusion.^21^ Recently, fabricating ionomers with porous structures has shown significant advantages in reducing *R*_local_.^22–24^ Using intrinsic microporous ionomers resulted in fast mass transport of gases and products and contributed to a high Pt mass-specific peak power density of 6.4 W mg_Pt_^−1^ at an ultra-low Pt loading of 0.07 mg cm^−2^.^25^ To achieve an extremely low *R*_local_, covalent organic frameworks (COFs), crystalline porous materials, have been introduced in ionomers and exhibit a significant improvement in fuel cell performance and PGM utilization rate with low *R*_local_. Feng^26^ reported a COF-based ionomer that tailored the three-phase microenvironment with mesoporous apertures of 2.8 to 4.1 nanometers and appendant sulfonate groups, promoting both proton transfer and oxygen permeation. As a result, the PEMFC exhibited 1.6 times the peak power density compared to those without the COF. Building on the excellent performance of COF-based ionomers in promoting proton and oxygen transfer, Feng^27^ developed a “breathable” proton conductor by combining α-aminoketone-linked COF ionomers with Nafion. This combination leverages synergistic hydrogen bonding to retain water, enhancing hydration and proton transport, while reducing oxygen transport resistance. The resulting PEMFC could operate under 105 °C with a superhigh peak power density (18.1 and 9.5 watts per milligram of Pt) and showed increases of 101% (under H_2_–O_2_) and 187% (under H_2_-air), compared with cells without the COF. These studies confirmed that COF materials played an important role in reducing oxygen transport resistance. The ionomer not only is used to reduce oxygen transfer resistance, but also serves as a binder to anchor catalyst particles and plays a crucial role in constructing the principal catalyst layer triple-phase boundary microenvironment.^28–30^ However, COF powder is insoluble and could not bind the catalyst particles, necessitating the addition of Nafion as a binder to form a uniform three-phase boundary with the catalyst for these reported COF-based ionomers.

To develop COF-based ionomers combining low oxygen transport resistance and binding functionality, we fabricated colloidally dispersed COF-polymer ionomers *via* chemical bonding of COF particles with hyperbranched polymer (B-TPPT) or linear polymer (PTP). The COF-polymer ionomers as a binder exhibited a uniform distribution of catalyst particles and excellent binding properties, facilitating the formation of a uniform three-phase boundary with microporous structure coating on the surface of catalysts. The COF-polymer electrode achieved a 96.4% reduction in *R*_local_ compared to the polymer electrode without COF materials. Given the limited research on alkaline ionomers, we chose anion exchange membrane fuel cells to evaluate the performance of these ionomers. Under ultra-low Pt loading (60 μg cm^−2^), a fuel cell with the COF-polymer ionomer exhibited a peak power density of 0.78 W cm^−2^, which was 3 times that of the polymer electrode without COF.

## Results and discussion

### Synthesis and characterization

**Fig. 1:**
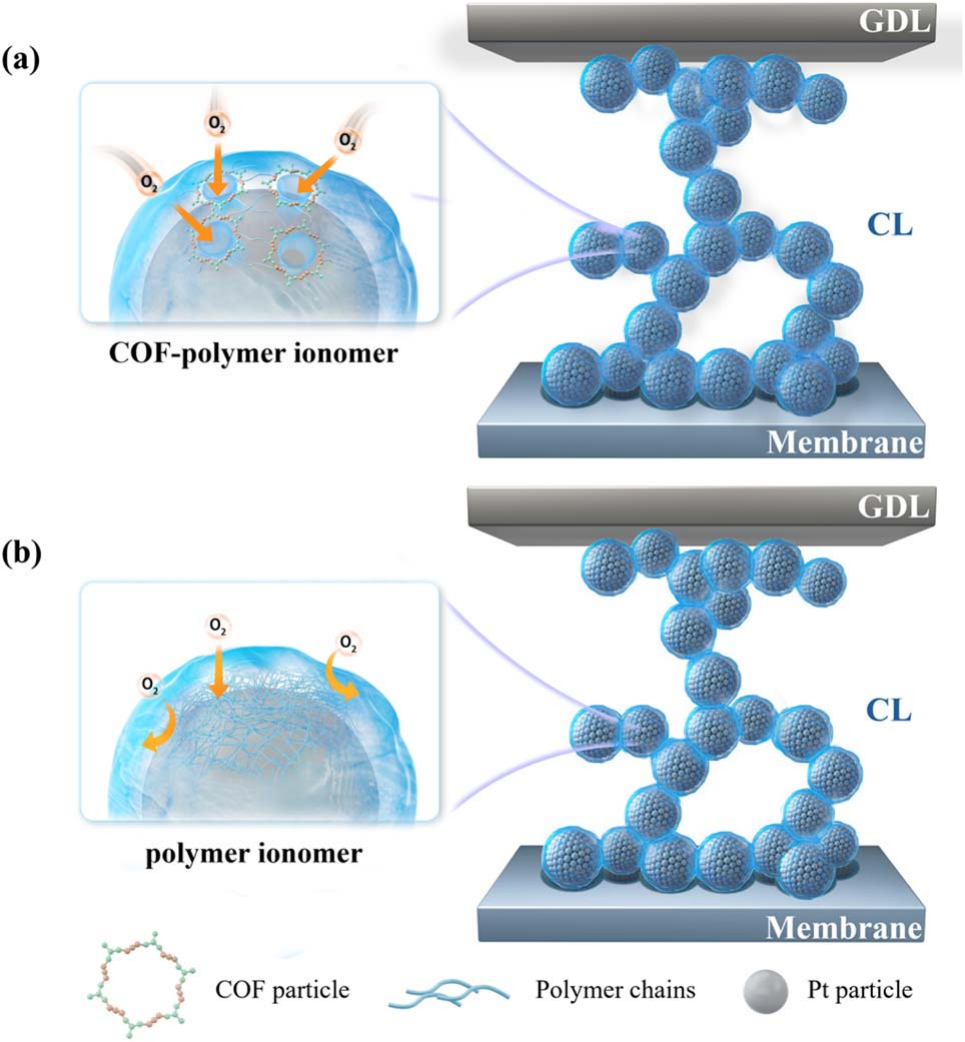
Schematic diagram of local oxygen transfer in CL with (a) COF-polymer ionomer and (b) polymer ionomer.

**Fig. 2:**
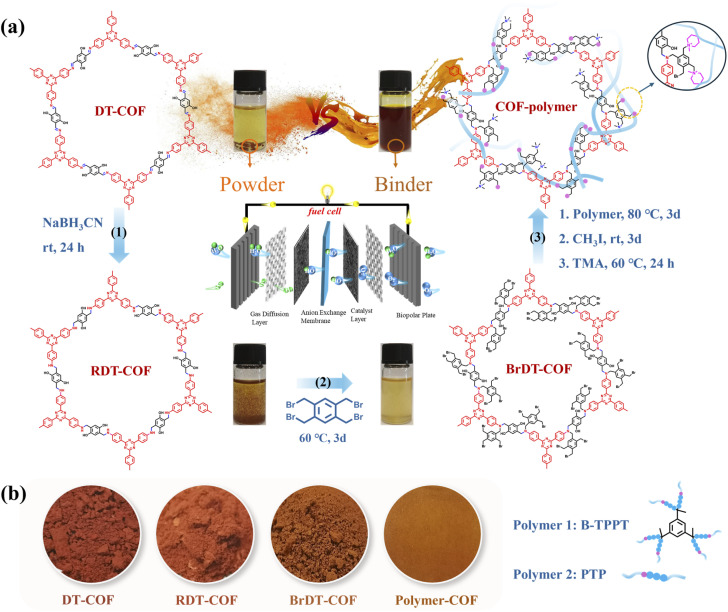
(a) The synthesis route of COF-polymer ionomers and photographs of the solubility of DT-COF, RDT-COF, BrDT-COF and COF-polymer (B-TPPT-COF) in DMSO. (b) Photographs of DT-COF powder, RDT-COF powder, BrDT-COF powder and COF-polymer (B-TPPT-COF) membrane; and schematic of B-TPPT and PTP polymer structures.

As shown in Fig. 1a, the introduction of a stable colloidal dispersion of COF-polymer ionomers resulted in the formation of a well-defined microporous structure on the surface of the catalysts. This microporous architecture effectively provided an efficient pathway for local oxygen transfer to the catalyst, particularly in the complex three-phase microenvironment, where solid, liquid, and gas phases interact. The microporous structure facilitated the diffusion of oxygen molecules to the active sites of the catalyst, significantly reducing the local resistance to oxygen transport (*R*_local_) and thereby optimizing the rate of the ORR and improving the fuel cell efficiency. As shown in Fig. 1b, in contrast, a traditional polymer ionomer, which lacks the COF material, typically forms a dense and tightly wound layer around the catalyst surface. This compact structure creates a physical barrier that hinders the effective transport of oxygen to the Pt active sites. As a result, oxygen diffusion becomes significantly slower, leading to increased *R*_local_. The restricted oxygen availability negatively impacts the ORR rate, limiting the efficiency and overall performance of the fuel cell. Thus, the incorporation of COF materials into polymer ionomers represents a significant improvement over traditional ionomer designs, offering better oxygen transport, enhanced catalytic activity, and more efficient utilization of the catalyst's surface area in ORR applications. Specifically, COF-polymer ionomers were synthesized *via* an alkylation reaction, which chemically bonds COF particles with the polymer chains. As illustrated in Fig. S1, DT-COF was prepared from its monomers, 1,3,5-tris(4-amidophenyl)triazine (TA) and 2,5-dihydroxyterephthaldeyde (DH), *via* solvothermal treatment. This crystalline COF powder was insoluble in common solvents (*e.g.*, DMSO, as shown in Fig. 2a). To achieve a colloidally dispersed COF binder, further functionalization was required. As illustrated in Fig. 2a(1), the –C=N– group in DT-COF was reduced to –C–NH– by NaBH_3_CN at room temperature, yielding RDT-COF. This reduction not only improved the alkaline stability of RDT-COF,^31^ but also introduced reaction sites for further functionalization. The –C–NH– group in RDT-COF was reactive and easily attacked by –Br for the Hofmann alkylation reaction. Thus, BrDT-COF was obtained through the reaction of RDT-COF with 1,2,4,5-tetrakis(bromomethyl)benzene (Fig. 2a(2)). As shown in Fig. 2a(3), COF-polymer binders (B-TPPT-COF and PTP-COF) were prepared *via* alkylation of the bromine group of BrDT-COF with the tertiary amine group of the polymers (the chemical structures of B-TPPT and PTP polymers are shown in Fig. S2), followed by a Menschutkin reaction with CH_3_I and an alkylation reaction with trimethylamine (TMA), respectively. Because of the grafted polymer chains, COF-polymer ionomers exhibited excellent solubility in DMSO compared to the un-grafted COF particles, as shown in Fig. 2a. Fig. 2b shows that the red powder of DT-COF turned orange (BrDT-COF) after functionalization. After these three modification steps, the insoluble crystalline COF powder was transformed into a colloidally dispersed COF-polymer ionomer, which exhibited excellent film-forming properties.

**Fig. 3:**
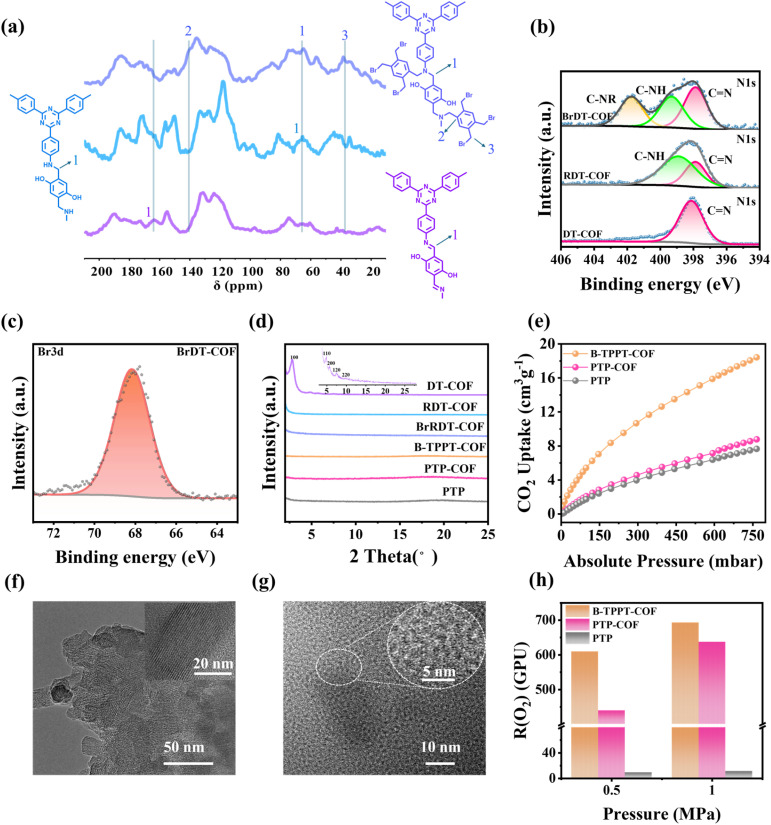
(a) ^13^C solid-state NMR spectra and (b) XPS survey spectra of N element of DT-COF, RDT-COF and BrDT-COF. (c) XPS survey spectrum of Br element of BrDT-COF. (d) XRD patterns and (e) CO_2_ uptake of B-TPPT-COF, PTP-COF and PTP. TEM images of (f) DT-COF and (g) B-TPPT-COF. (h) The O_2_ permeation rate of B-TPPT-COF, PTP-COF and PTP (1 GPU = 10^−6^ cm^−2^ s^−1^ cm Hg^−1^).

^13^C solid-state nuclear magnetic resonance (^13^C ssNMR) spectra were measured to confirm the chemical structures of DT-COF, RDT-COF and BrDT-COF powders. As shown in Fig. 3a, the characteristic signal at 165 ppm in the purple line was attributed to the chemical shift of the C=N bond in DT-COF powder.^32^ After modification, this peak significantly weakened, and new peaks appeared at 65 ppm for RDT-COF powder (blue line) and at 140 ppm and 38 ppm for BrDT-COF (bluish violet line), corresponding to the chemical shifts of the C–N, C–Ph and C–C bonds, respectively. The Fourier transform infrared spectroscopy (FTIR) results revealed a decrease in the peak intensity at 1580 cm^−1^ (assigned to C=N stretching) and an increase at 804 cm^−1^ (attributed to C–N bending), confirming the partial conversion of C=N to C–N (Fig. S3).^33^ These results indicate successful modification. Additionally, Fig. 3b shows the X-ray photoelectron spectroscopy (XPS) results for the nitrogen element in DT-COF, RDT-COF and BrDT-COF. A peak at 398 eV corresponded to the C=N bond (red area). After modification, new peaks at 399 eV and 402 eV appeared, corresponding to C–NH (green area) in RDT-COF and C–NR (yellow area) in BrDT-COF. Additionally, a Br–C peak appeared at 67.05 eV in Fig. 3c for BrDT-COF, confirming the successful grafting of 1,2,4,5-tetrabromomethylbenzene onto RDT-COF. The ^13^C ssNMR, FTIR, and XPS results confirmed the successful fabrication of DT-COF, RDT-COF and BrDT-COF. Fig. S4 exhibits the thermal stability data of the COF materials. DT-COF did not show significant degradation until 450 °C, while RDT-COF and BrDT-COF began to lose weight at 180 °C. This was due to the reduction of the C=N bond, producing alkyl side chains that degraded below 200 °C. Scanning electron microscopy (SEM) images in Fig. S5 show that the COF particles maintained their porous structure before and after modification. The chemical structure of the COF-polymers was confirmed *via*^1^H NMR (Fig. S6). X-ray diffraction (XRD) was conducted to explore the structure of the COF powders and COF-polymers. Fig. 3d shows that DT-COF exhibited high crystallinity, with characteristic diffraction peaks at 2.76°, 4.81°, 5.71°, 7.45° and 9.82° in the PXRD pattern, corresponding to the (100), (110), (200) and (210) reflection planes.^32^ However, after the C=N bond was modified to a C–N bond, the crystalline structure became disordered due to the conformational flexibility of the secondary amine linkages,^26^ and no crystallization signals were observed in the XRD patterns of RDT-COF, BrDT-COF and COF-polymers. N_2_ absorption measurement and transmission electron microscope (TEM) images further revealed the structure of the COF powders and COF-polymers. DT-COF exhibited a BET surface area of 1579.95 m^2^ g^−1^ (purple line in Fig. S7a), with a pore size of 3.62 nm (Fig. S7b), and an ordered lattice streak in TEM images (Fig. 3f). Due to the disruption of the crystalline structure, the BET surface area of the modified COF materials and COF-polymers decreased significantly (Fig. S7a), and the ordered lattice streaks disappeared (Fig. S8a and b). TEM images of the COF-polymers in Fig. 3g and S8c show some nano-scaled black areas with few preserved crystalline zones (enlarged portion in the white circle of Fig. 3g). These black zones corresponded to COF particles, with sizes of around 50 nm, suggesting that chemical bonding effectively enhanced the compatibility between insoluble and rigid BrDT-COF particles and the soft polymer chains, resulting in an excellent nanoscale dispersion. SEM images of the COF-polymers in Fig. S9 further confirmed this good compatibility. After introduction of COF materials, the hyperbranched B-TPPT-COF demonstrated significantly higher CO_2_ uptake (18.4 cm^3^ g^−1^, STP) as shown in Fig. 3e and S10a, while PTP-COF (8.8 cm^3^ g^−1^, STP) also exhibited increased CO_2_ uptake compared to PTP (7.6 cm^3^ g^−1^, STP). To confirm the capacity for O_2_ permeation of B-TPPT-COF, PTP-COF and PTP, the O_2_ permeability of B-TPPT-COF, PTP-COF and PTP membranes was measured at 80 °C and 0% relative humidity (RH). As illustrated in Fig. 3h, compared to the PTP membrane without COF materials, the B-TPPT-COF and PTP-COF membranes showed higher O_2_ permeability, indicating that the introduction of BrDT-COF effectively created a micro-porosity in the polymers (Fig. S10b), enhancing O_2_ permeation. The B-TPPT-COF membrane exhibited the highest O_2_ permeability, which can be attributed to the hyperbranched structure of B-TPPT-COF, leading to a higher free volume than that of the linear structure of the PTP-COF membrane.

The COF-polymer ionomer exhibited excellent membrane-forming ability and these properties are shown in Fig. S11. As shown in Fig. S11a, for a small difference in ion exchange capacity (IEC), the water uptake of PTP-COF (2.1 g mmol^−1^) at 80 °C was significantly higher (258.6%) than that of PTP (2.6 g mmol^−1^, 122.2%), which may be attributed to the rich porous structure introduced by the COF framework, providing more space to accommodate water molecules. However, B-TPPT-COF (1.65 g mmol^−1^) exhibited a lower water uptake (86.5% at 80 °C), resulting from its considerably reduced IEC. The swelling ratio followed a similar trend to water uptake. As shown in Fig. S11b, the PTP-COF membrane had a higher swelling ratio (24.7%) than the PTP (16.8%) and B-TPPT-COF membranes (11.4%) at 80 °C. As shown in Fig. S11c, due to its high IEC and water uptake, the PTP-COF membrane achieved high hydroxide conductivity (213.5 mS cm^−1^ at 80 °C). Although the IEC of the B-TPPT-COF membrane was only 1.65 g mmol^−1^, its porous structure and high free volume allowed it to achieve good hydroxide conductivity of approximately 123 mS cm^−1^ at 80 °C, which meets the requirements for fuel cell application. The mechanical properties of these COF-polymer ionomers were affected by the introduction of COF materials. As shown in Fig. S11d, the strengths of the B-TPPT-COF (13.5 MPa) and PTP-COF (12.8 MPa) membranes were lower than that of the PTP membrane (28 MPa) in the wet state. As shown in Fig. S12, the alkaline stability of B-TPPT-COF was evaluated under harsh conditions (6 M KOH, 80 °C). Significant degradation was observed after 196 h of treatment. However, this level of chemical decomposition does not necessarily compromise its function as an ionomer in fuel cell applications.

### Structure and morphology

**Fig. 4:**
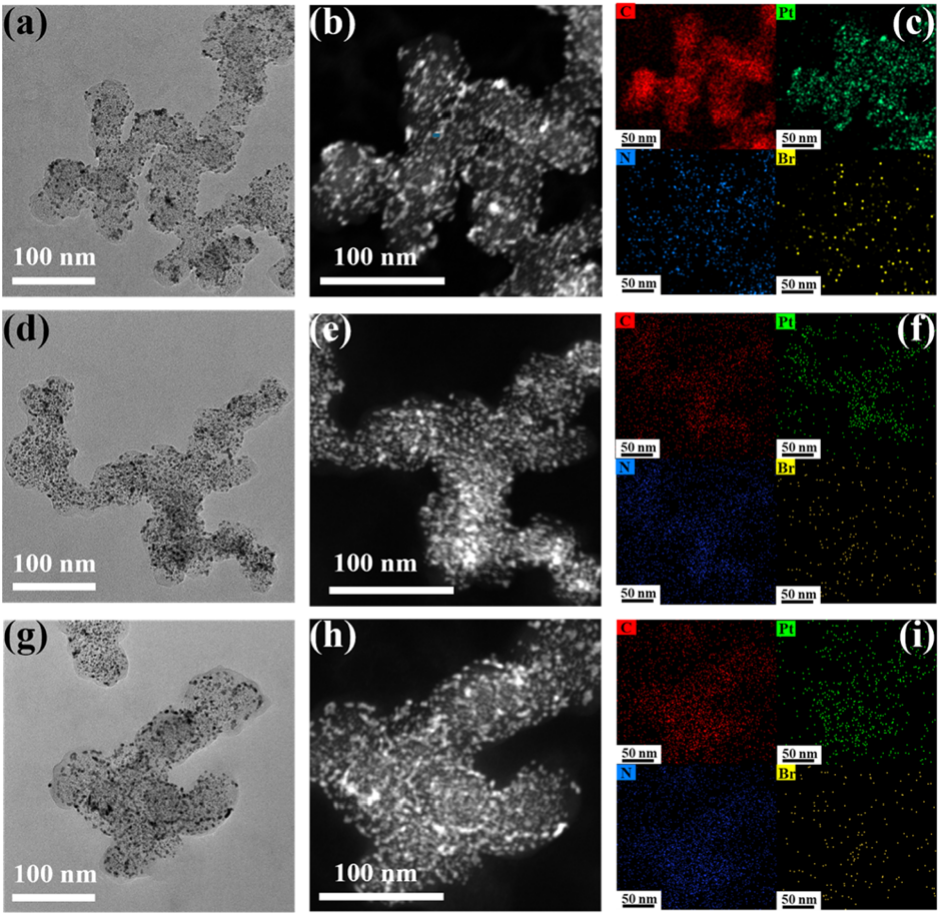
TEM images and EDS plots of catalyst inks with (a–c) B-TPPT-COF binder, (d–f) PTP-COF binder and (g–i) PTP binder.

TEM images of catalyst particles with B-TPPT-COF, PTP-COF and PTP ionomers are shown in Fig. 4. As shown in Fig. 4a, d and g, the catalyst particles with these three ionomers exhibited a homogeneous distribution. The black areas represent the Pt-based catalyst, while the surrounding gray areas correspond to a mixture of carbon support and binder. This distribution was more distinct in the HAADF images. As shown in Fig. 4b and e, compared to the PTP ionomer (Fig. 4h), Pt particles with B-TPPT-COF and PTP-COF binders formed small aggregates (bright white area), likely due to the introduction of COF particles. However, these aggregates were smaller than 7 nm and not of large scale, indicating that both B-TPPT-COF and PTP-COF ionomers provided good catalyst dispersion. This dispersion was primarily attributed to the chemical bonding between polymer chains and COF particles, which enhanced the interfacial compatibility of the insoluble COF particles with the polymer, resulting in good dispersion and adhesion for catalyst particles to construct a uniform three-phase boundary. These ionomers not only acted as bonding agents for the catalyst, but also ensured uniform coverage of the catalyst surface, reducing gas transfer resistance and improving catalytic activity. Energy dispersive X-ray spectroscopy (EDS) analysis of the C, N, Pt and Br elements was conducted to further investigate the distribution of the catalyst in the binder, with C, N, and Br originating from the binder. As shown in Fig. 4c, f and i, the EDS plots revealed that the elemental distributions of the catalyst and ionomer overlapped well, further confirming that the catalyst particles were uniformly distributed in the binders.

### Fuel cell performance

**Fig. 5:**
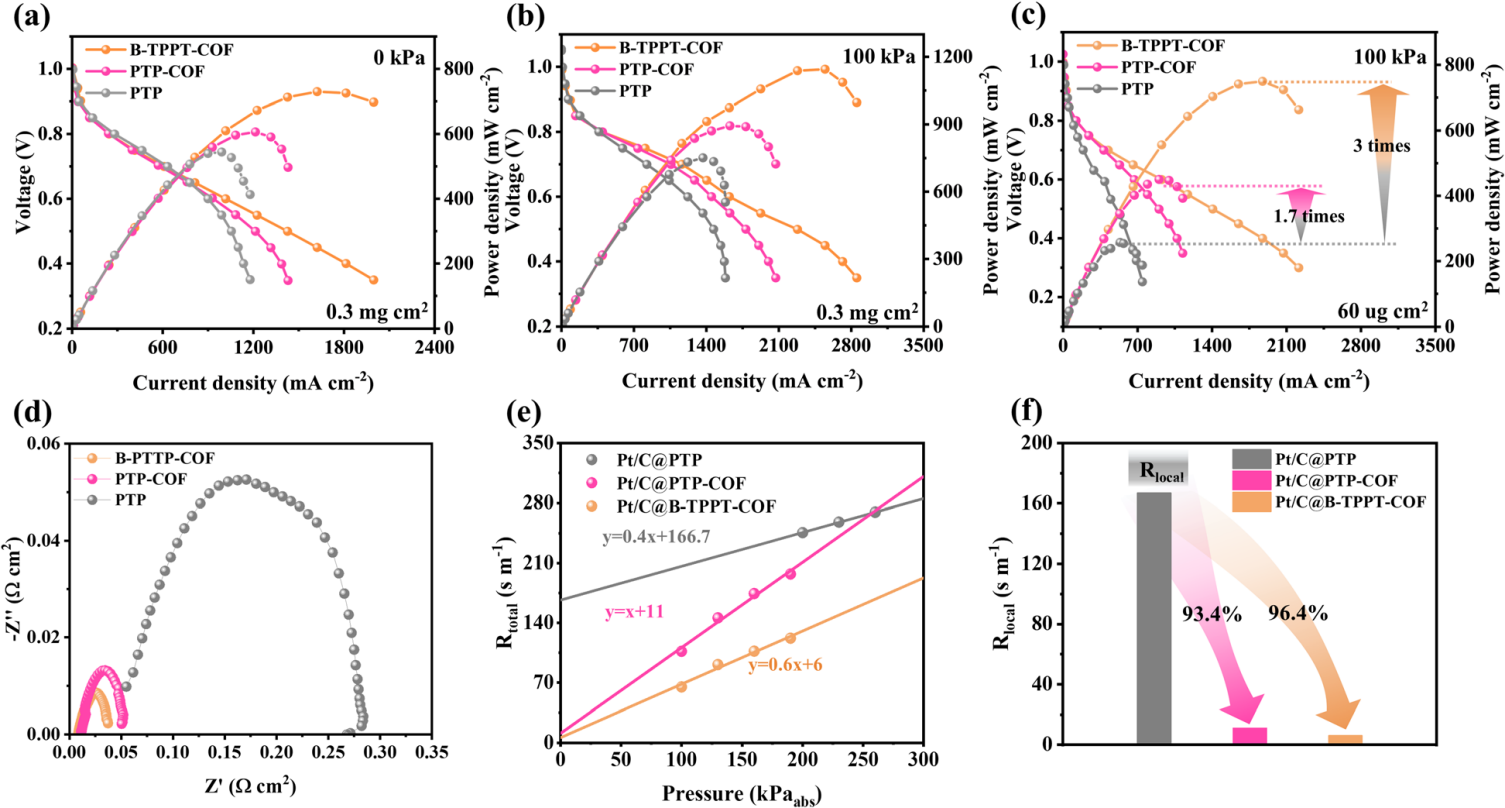
AEMFC performance based on B-TPPT-COF, PTP-COF and PTP binders at (a) 0 kPa, (b) 100 kPa at 80 °C with 0.3_Pt_ mg cm^−2^ loading of catalyst and (c) 100 kPa at 80 °C with 60_Pt_ ug cm^−2^ loading of catalyst. (d) Electrochemical impedance spectra at 700 mA cm^−2^ of B-TPPT-COF, PTP-COF and PTP electrodes. (e) Total oxygen transport resistance of B-TPPT-COF, PTP-COF and PTP electrodes as a function of absolute gas pressure at 80 °C and 100% RH and (f) *R*_local_ of B-TPPT-COF, PTP-COF and PTP electrodes.

To evaluate whether the COF-polymer ionomers could effectively manage gas flow in the CL during operation of AEMFCs, MEAs were prepared using B-TPPT-COF and PTP-COF as catalyst binders for both anode and cathode. A PTP binder without the COF materials was used for comparison. The MEAs were fabricated using the CCM (catalyst-coated membrane) method, in which iodomethane-treated B-TPPT was used as the AEM (20–30 μm). Pt/C and PtRu/C were adopted as cathode and anode catalysts, respectively, with a catalyst loading of 0.3_Pt_ mg cm^−2^. The polarization and power density curves of these MEAs were measured under H_2_–O_2_ conditions, as shown in Fig. 5. The open circuit voltages of all three MEAs were approximately 1 V at 100% RH, indicating low fuel crossover. As shown in Fig. 5a, the B-TPPT-COF electrode and PTP-COF electrode achieved peak power densities of ∼0.73 W cm^−2^ and 0.59 W cm^−2^ (at 0 kPa), respectively, higher than that of the PTP electrode (∼0.52 W cm^−2^ at 0 kPa). As back pressure increased, MEA with COF-polymer ionomers showed greater improvements compared to the PTP electrode. Under 100 kPa, as shown in Fig. 5b, the B-TPPT-COF electrode reached a peak power density of ∼1.14 W cm^−2^, 1.52 times that of the PTP electrode, and the PTP-COF electrode also exhibited a high peak power density (0.89 W cm^−2^ at 100 kPa) compared to the PTP electrode. As aforementioned, the O_2_ permeability of B-TPPT-COF and PTP-COF was significantly higher than that of PTP, suggesting that COF incorporation introduced a more porous structure in the COF-polymers. These results indicated that the porous structure of the COF-polymer enhanced the mass transition through the ionomer film that covered the catalyst surface. Additionally, the hyperbranched structure of the polymer backbone in B-TPPT-COF provided a high free volume, further reducing oxygen transfer resistance. Consequently, the MEA with B-TPPT-COF ionomer exhibited optimal fuel cell performance. The effect of reduced oxygen transfer due to the porous structure of the COF-polymer ionomer was particularly pronounced under ultra-low Pt loading. As illustrated in Fig. 5c, the B-TPPT-COF electrode achieved a peak power density of 0.78 W cm^−2^ with 60_Pt_ μg cm^−2^, corresponding to a mass-specific power density of 6.5 W mg^−1^, which is 3 times that of the PTP electrode. The PTP-COF electrode also achieved 1.7 times the peak power density of the PTP electrode. These results provided direct evidence that the COF-polymer ionomers, especially hyperbranched COF-polymer ionomers, facilitated oxygen transfer through the film to the catalyst.

As shown in Fig. 5d, electrochemical impedance spectroscopy (EIS) measurements under H_2_–O_2_ conditions reveal that incorporating porous COF structures promotes oxygen transport, thereby reducing the charge transfer resistance (*R*_ct_). Moreover, compared to PTP without COF materials, the ohmic resistance is also significantly decreased. These results indicate that the rigid framework of the COF may optimize the distribution of the ionomer, thereby enhancing the conduction pathways within the cell. Furthermore, the incorporation of porous COF materials exposes more active sites, which facilitates the electrochemical reaction and thus enables superior fuel cell performance even under low-Pt-loading conditions. To evaluate *R*_local_ in these electrodes, the limiting current method was used under a 1.0% O_2_ feed in the fuel cell at different back pressures. The recorded polarization curves under varying pressure for the B-TPPT-COF, PTP-COF, PTP and B-TPPT electrodes are shown in Fig. S13 and S14. It was evident that the COF-polymer electrodes exhibited higher limiting current density than both the PTP and B-TPPT electrodes. Notably, the B-TPPT-COF electrode achieved the highest limiting density at the same pressure, indicating superior local O_2_ permeability in the catalyst surface with COF-polymer ionomers. Fig. 5e and S14 showed the total resistance (*R*_total_) for these four electrodes, which represented the O_2_ transport resistance in the CL. This was calculated using pressure-dependent molecular diffusion, with the intercept corresponding to *R*_local_ of the CL. As illustrated in Fig. 5f and S14, *R*_local_ of B-TPPT-COF and PTP-COF electrodes decreased significantly to be 11 s m^−1^ and 6 s m^−1^, respectively, compared to B-TPPT ( 29.0 s m^−1^) and PTP (166.7 s m^−1^). The substantial reduction of 93.4% and 96.4% in *R*_local_ for COF-polymer ionomers indicated that the introduction of porous COF materials significantly improved local O_2_ diffusion on the Pt surface, thereby enhancing catalytic activity. This was the primary reason for the superior performance of the B-TPPT-COF and PTP-COF electrodes.

## Experimental

### Materials

1,3,5-Tris(4-amidophenyl)triazine (TA, 98%) and 2,5-dihydroxyterephthaldeyde (DH, 98%) were purchased from Jilin Zhongke Science and Technology Co. Sodium cyanoborohydride (NaBH_3_CN) was purchased from Aladdin. 1,2,4,5-Tetrakis(bromomethyl)benzene was purchased from Sigma-Aldrich. Trimethylamine (25% in water) 1-butanol, tetrahydrofuran (THF), 1,2-dichlorobenzene, *N*,*N*-dimethylacetamide (DMAC), dimethyl sulfoxide (DMSO), *N*,*N*-dimethylformamide (DMF) and other chemical solvents were purchased from Adamas. All chemical regents and solvents were used as received without further purification.

### Synthesis of DT-COF powder

In a pressure-resistant vial, TA (0.225 g, 0.63 mmol), DH (0.159 g, 0.8 mmol), 1,2-dichlorobenzene (45 mL), 1-butanol (45 mL) and aqueous 6 M acetic acid (9 mL) were added. After the suspension was ultrasonicated for 15 min, the vial was flash-frozen in a liquid N_2_ bath and degassed by three freeze–pump–thaw cycles. The vial was sealed off and then heated to 120 °C for 72 h. After the reaction, the suspension was cooled to room temperature, filtered and washed 3 times with DMAC, ethanol and acetone, respectively. A red–orange powder (DT-COF) was collected by filtration and vacuum-dried at 80 °C for 24 h.

### Synthesis of RDT-COF powder

In a flask, DT-COF (0.2 g, 1.02 mmol imine), anhydrous THF (250 mL), and glacial acetic acid (700 μL, 12.2 mmol) were added. After the mixture was stirred for 10 min, NaBH_3_CN (21 mL, 21 mmol) was drawn into the flask with a syringe within 20 min. The system was reacted at room temperature for 24 h. Then, an orange fluffy powder was collected by filtration and washed 3 times each with deionized water and ethanol, followed by drying in an oven at 80 °C, the product being named as RDT-COF.

### Synthesis of BrDT-COF powder

In a flask, RDT-COF (1 g, 0.005 mol of –NH–), 1,2,4,5-tetrabromomethylbenzene (9 g, 0.02 mol), potassium carbonate (K_2_CO_3_, 3.45 g, 0.025 mol), potassium iodide (KI, 0.83 g, 0.005 mol) and solvent DMSO (10 mL) were added. The reaction was carried out at 60 °C in an oil bath for 48 h. Then the suspension was centrifuged and washed several times with DMSO and deionized water until the potassium salt and unreacted monomer were washed out. The dark orange fluffy powder, BrDT-COF, was obtained by drying in an oven at 60 °C.

### Synthesis of colloidally dispersed COF ionomer (B-TPPT-COF)

The B-TPPT and PTP copolymers were synthesized *via* a previously reported method.^34^ Additionally, based on previously established optimal ratios and ionomer-specific requirements, a selected ratio was used for further investigation.^35^ In a flask, BrDT-COF (0.0048 g), polymer B-TPPT (0.1 g) and solvent *N*-methylpyrrolidone (4 mL) were added, and the system was reacted at 80 °C for 72 h. After the reaction was cooled down to room temperature, CH_3_I (1 mL) was added and this mixture was reacted for another 48 h in a dark environment. Then the reaction was precipitated in ethyl acetate and followed by filtering. The obtained polymer was immersed in an aqueous trimethylamine solution at 60 °C for 24 h, followed by drying at 80 °C to yield B-TPPT-COF. The PTP-COF ionomer was synthesized using the above procedure.

### Preparation of B-TPPT-COF ionomer solution

B-TPPT-COF (50 mg) was added in DMSO (800 μL), heated to dissolve and followed by the addition of ethanol (200 μL) to obtain a 5% solution of the ionomer.

## Conclusion

In summary, we have fabricated two stable colloidal dispersions of polymer-grafted COF ionomers (B-TPPT-COF with a hyperbranched polymer and PTP-COF with a linear polymer) by chemical bonding of polymer chains with COF materials. TEM and SEM images revealed the uniform dispersion of rigid COF particles in the polymers, confirming that the chemical bonding method successfully enhanced the compatibility between the COF particles and the polymer chains. Compared to the PTP ionomer (11.5 GPU, without COF materials), both B-TPPT-COF (693 GPU) and PTP-COF (637 GPU) ionomers exhibited significantly higher O_2_ permeability due to the introduction of porous COF particles. TEM images of catalyst inks indicated that the porous COF-polymers could uniformly coat the catalyst surface, effectively binding catalyst particles to form a uniform three-phase boundary. EIS results confirmed that the MEA with COF-polymer ionomers (B-TPPT-COF and PTP-COF) established a more efficient hydroxide transport pathway. The limiting current measurement confirmed that these COF-polymer ionomers significantly reduced *R*_local_ of O_2_ in the CL. Particularly under ultra-low Pt loading, the enhanced local O_2_ diffusion allowed the B-TPPT-COF electrode to achieve a peak density of 0.78 W cm^−2^ under H_2_–O_2_ conditions, three times that of the PTP electrode (0.26 W cm^−2^). This study presented a method for fabricating stable colloidal dispersions of COF-polymer ionomers, which compensated for the drawback that COF materials cannot bind catalyst particles and emphasized the role of COF materials in reducing local O_2_ diffusion resistance in the CL, offering an alternative approach to more efficient ionomers for fuel cells.

## Author contributions

Xiaoqin Ma, Xiaoli Lu, Shimei Liang, Jianchuan Wang and Zidong Wei conceived and designed the experiments; Xiaoqin Ma and Shimei Liang finished most of the experiments; Xiaoqin Ma, Xiaoli Lu, Jianchuan Wang and Zidong Wei completed the writing; Caili Yuan prepared the TEM samples; Jingtao Si analyzed the TGA data; Jianchuan Wang and Zidong Wei directed the project.

## Conflicts of interest

There are no conflicts to declare.

## Supplementary Material

SC-016-D5SC04070A-s001

## Data Availability

The authors declare that the data supporting the findings of this study are available within the main text and in the supplementary information (SI), or from the corresponding author on reasonable request. Supplementary information: includes the characterization, and Fig. S1–S14. See DOI: https://doi.org/10.1039/d5sc04070a.
