# Supplementary material for: Achieving ultra-low oxygen transport resistance of fuel cells by microporous covalent organic framework ionomers
Source: Chem Sci. 2025 Oct 6;16(46):22111–8. doi: 10.1039/d5sc04070a (PMC12542847; doi:10.1039/d5sc04070a)
Supplement: SC-016-D5SC04070A-s001 [file SC-016-D5SC04070A-s001.pdf]

Supplementary Information (SI) for Chemical Science.
This journal is © The Royal Society of Chemistry 2025

Supporting Information

Achieving Ultra-low Oxygen Transport Resistance of Fuel Cell by Microporous Covalent Organic Framework Ionomers

Xiaoqin Ma[1,2†], Xiaoli Lu[1†], Shimei Liang[1†], Caili Yuan[1], Jingtao Si[1], Jianchuan Wang[1] and Zidong Wei[1]*

[1] School of Chemistry and Chemical Engineering, State Key Laboratory of Advanced Chemical Power Sources, Chongqing University, 400044 Chongqing, China

[2] Northwest Electric Power Design Institute Co., Ltd, of China Power Engineering Consulting Group, Xi'an, 710075, shaanxi Province, China

Email: jxw319@cqu.edu.cn

Keywords: Covalent Organic Framework, COF, Ionomer, Oxygen transport, Fuel cells

Characterization

The ^{1}H spectra of samples in OH^- form were measured by an Agilent 400 MR DD2 spectrometer using DMSO-d6. The instruments used to measure the surface and cross-section morphology in this paper was JEOL JSM-5900LV and the test voltages are selected to be 10 kV and 20 kV. Energy-dispersive X-ray spectroscopy (EDS) were taken on the same microscope. Sample preparation: membrane samples were fractured in liquid nitrogen, powder samples were dispersed in water by ultrasonic, and then 10 uL of the well-dispersed solution was added onto a clean silicon wafer surface, followed by drying under an infrared lamp. Samples sputtered with gold before analysis. Transmission electron microscopy (TEM, JEOL JEM-200CX TEM, 200kV) was used to analyze the morphology of ionomer samples. Sample preparation: 10 uL of the sample dispersion was dropped onto the copper mesh and dried under the infrared lamp before test. Energy-dispersive X-ray spectroscopy (EDS) were taken on the same microscope. The thermal properties of samples were measured by a thermogravimetric analyzer (TGA-Q500, TA Instruments) under N_2 atmosphere. Samples were hold at

100 °C for 3 min to remove adsorbed water, and then heated from 100 °C to 1000 °C with a heating rate of 10 °C min^{-1}. The specific surface area of the sample was measured by the method at liquid nitrogen temperature using an instrument Kubo-X1000 surface area analyzer. Before testing, the bulk samples were ground into powder as much as possible, and the samples were first evacuated under dynamic vacuum at 120°C overnight to remove the guest molecules and adsorbed moisture before re-measuring the accurate sample weight. X-ray diffraction (XRD, Empyrean) analyses using Cu-Kα X-ray radiation (λ = 0.154056 nm) with 50 kV voltage and 55 mA current. Samples were mounted onto silicon zero background holder. The XRD patterns were recorded from 1° to 40° (2θ) with a step size of 0.02 degree and a scan rate of 10° min^{-1}. X-ray photoelectron spectroscopy (XPS, Thermo Kalpha) using 200 W monochromated Al-Ka radiation. The binding energies were referred to the C1s peak (284.8 eV) from adventitious carbon. The gas permeabilities of B-TPPT-COF, PTP-COF and PTP (20 μm) were measured by a gas penetration test apparatus (Agilent 7890B). O_2 (0% relative humidity, and the flow rate of the test gas was 0.6 L/min) was used as testing gas at 80°C under different testing pressure (50 KPa and 100 KPa).

IEC measurement

The ion exchange capacity (IEC) of COF-polymer (B-TPPT-COF, PTP-COF) and PTP samples was measured by titration method. Before testing, the I^- of ~ 0.2g samples was exchanged into OH^- with 1 M KOH for 48 h, then, the residual KOH of these membranes were removed with deionized water. These membranes (OH^- form) were soaked in HCl solution (0.1 M, 50 ml) at room temperature for 48 h, followed by titration with 0.1 M NaOH and repeated three times. At last, weights (W_{dry}) of samples (Cl^- form) were recorded after drying in an oven under vacuum at 80 °C overnight. The IEC (mmol g^{-1}) of B-TPPT-COF, PTP-COF and PTP can be calculated by:

$$IEC = \frac{C_{HCl} V_{HCl} - C_{NaOH} V_{NaOH}}{W_{dry}}$$

Where V_{HCl} and V_{NaOH} were the volumes (ml) of HCl and NaOH, C_{HCl} and C_{NaOH} were the concentrations of HCl and NaOH.

Water uptake and swelling ratio

First, the W_{dry} (dry weight) and L_{dry} (dry length) of B-TPPT-COF, PTP-COF and PTP membranes (OH^- form) were recorded. Second, after these membranes were soaked in deionized water for 12 h at different temperature, wiping the water on the membrane surface and recorded the W_{wet} and L_{wet} of membranes.

The water uptake (WU) can be calculated by:

$$WU = \frac{W_{wet} - W_{dry}}{W_{dry}} \times 100\%$$

The swelling ratio (SR) can be calculated by:

$$SR = \frac{L_{wet} - L_{dry}}{L_{dry}} \times 100\%$$

In-plane OH^- conductivity

Impedance/gain-phase analyzer (Solatron 1260) was used to measure the in-plane OH^- conductivity of B-TPPT-COF, PTP-COF and PTP membranes, the frequency range of 1 to 10^7 Hz. The samples (OH^- form, without residual KOH) were clamped in the testing fixture and soaked in deionized water, then the Ohmic resistance was obtained from the associated Nyquist plot. The OH^- conductivity (σ) was calculated by:

$$\sigma = \frac{L}{RA}$$

Where R was the membrane resistance, L was the distance between the two electrodes, and A was the cross-sectional area of the membrane.

Full cell performance

The H_2-O_2 full cell performance of soluble COF-polymer ionomers were operated on a fuel cell test station (850e Multi Range, Scribner Associates Co.). The catalyst-coated membrane (CCM) method was adopted to prepare the MEA. Pt/C (60 wt.% in metal content) or PtRu/C (75 wt.% in metal content) catalysts were mixed with ionomer (B-TPPT-COF, PTP-COF and PTP) and isopropyl alcohol, ultrasonicated to form homogenous cathode or anode inks consisting of ionomer and catalyst (1:4). Then, the cathode and anode inks were sprayed onto the cathode and anode side of the membrane (B-TPPT), respectively. The metal loading at the anode and cathode were both 0.3 mg cm^{-2} or 60 μg cm^{-2}, with an effective electrode area of 5 cm^2. Subsequently, the as-prepared CCM was positioned between two carbon.

Testing of Limiting Current Density (i)

In this paper, the instrument used to test the limiting current density was the Model 850e fuel cell test system from Scribner Assoiates, U.S.A. The limiting current density test conditions were as follows: the cell temperature (T) was 80°C; the cathode gas humidification was 100% RH; the gas flow rates at the anode (H_2) and cathode (1% O_2:N_2=1:9) varied depending on the specific conditions, and the test voltage is as low

as 0.05 V, where a low concentration of oxygen is chosen to reduce the ultimate current density to ensure that there is no excess product water generation. Three or more pressures were selected for the back pressure during the test, and the corresponding limiting current density i was obtained by testing at different pressures, and the total transport resistance RT at different pressures was calculated by bringing i into Eq. $R_T = \frac{4F}{i_{lim}} \frac{P - P_W}{RT} X_{O_2}^{inlet}$. According to Eq. $R_T = \frac{4F}{i} \Delta C_{O_2} = R_{mol} \frac{P}{P_0} + R_{other}$, different R_T and pressures P were fitted to obtain the slopes and intercepts as R_{mol} and R_{other}, respectively, and when the catalyst Pt loading is extremely low, $R_{other} \approx R_{local}$.

Results

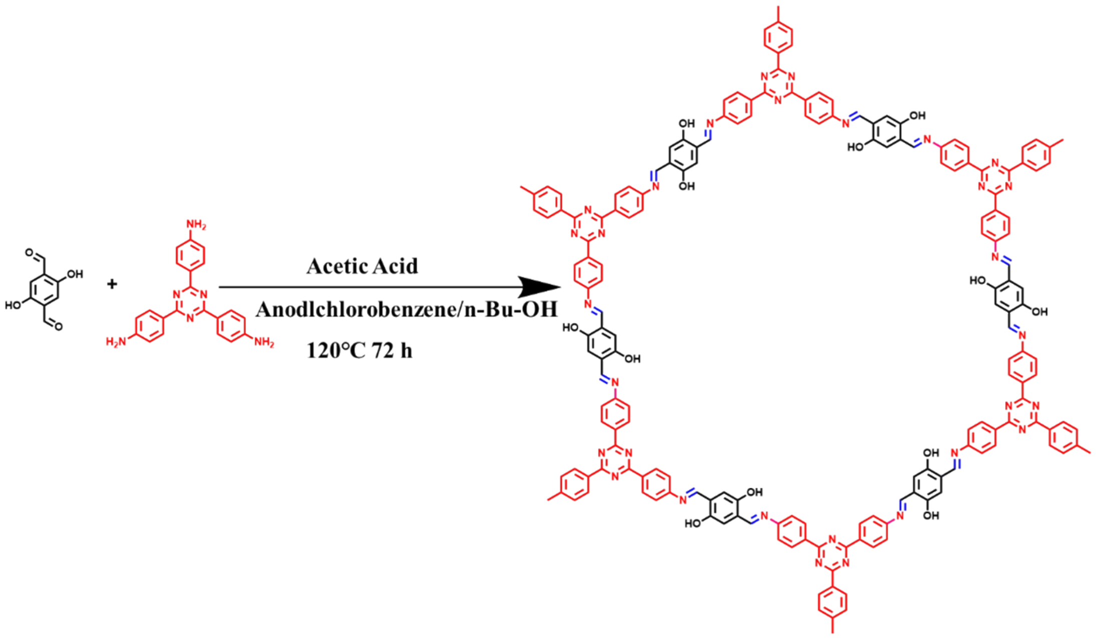

Figure S1 The synthesize route of DT-COF materials.

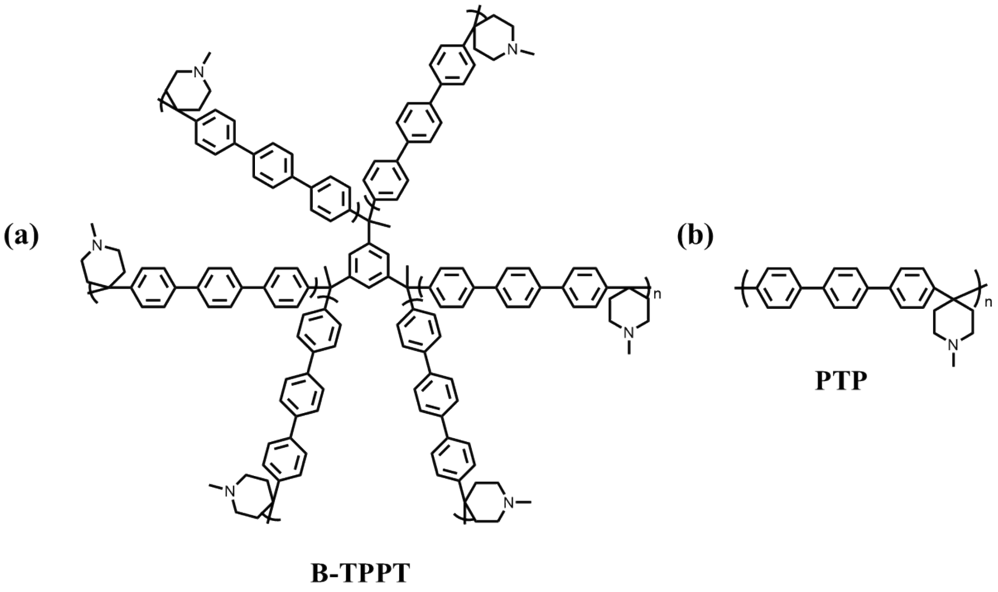

Figure S2 The chemical structure of (a) B-TPPT and (b) PTP.

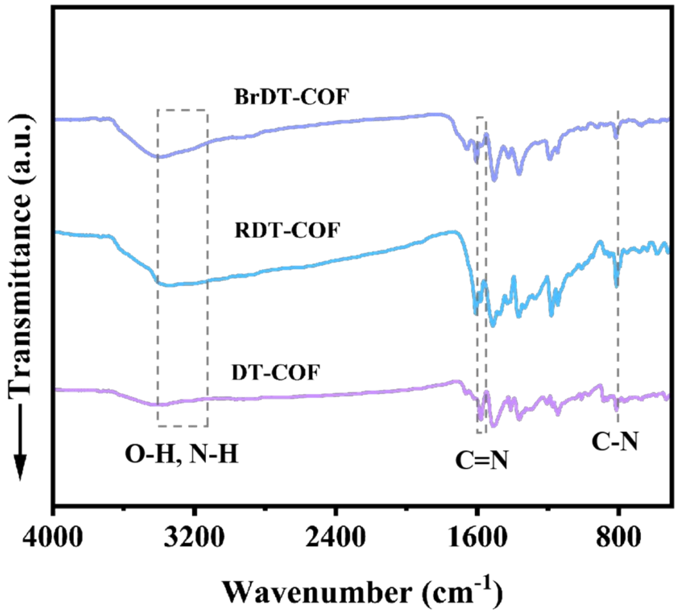

Figure S3 FT-IR spectra for DT-COF, RDT-COF and BrDT-COF.

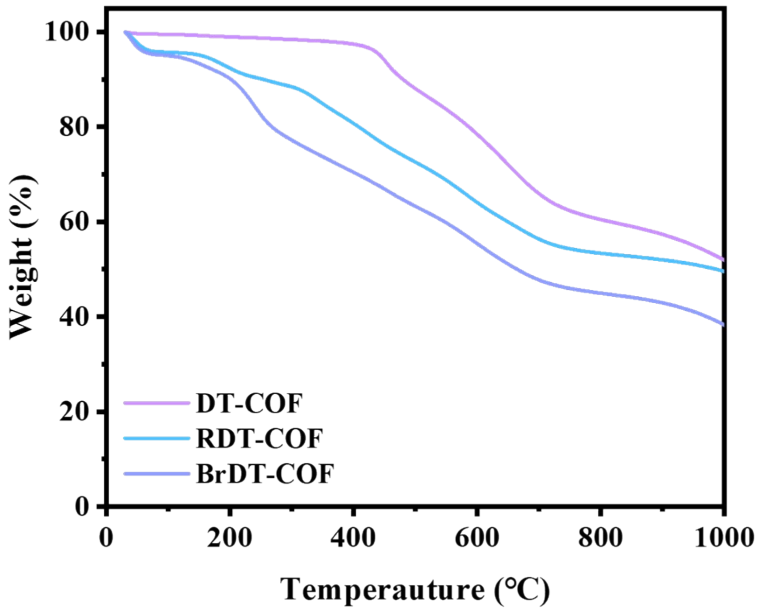

Figure S4 TGA curves of DT-COF, RDT-COF and BrDT-COF.

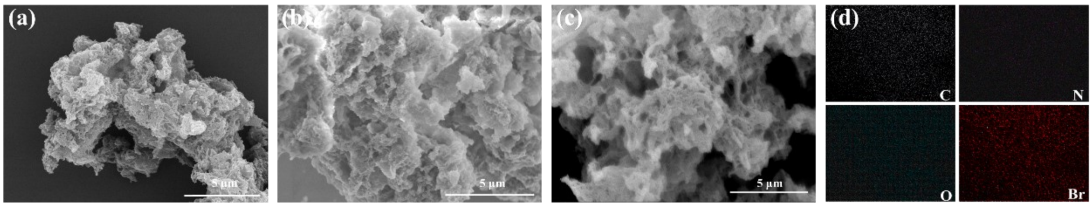

Figure S5 SEM images of (a) DT-COF, (b) RDT-COF and (c) BrDT-COF, (d) the EIS result of BrDT-COF.

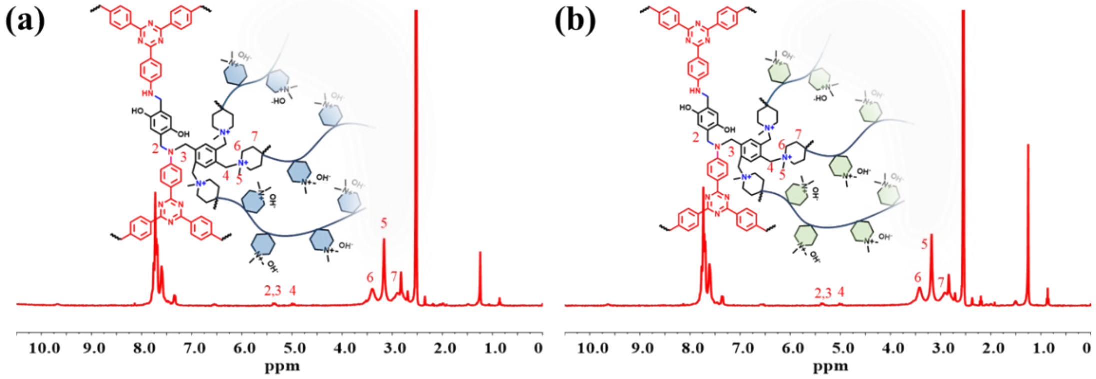

Figure S6 The ^{1}H NMR spectra of (a) B-TPPT-COF and (b) PTP-COF.

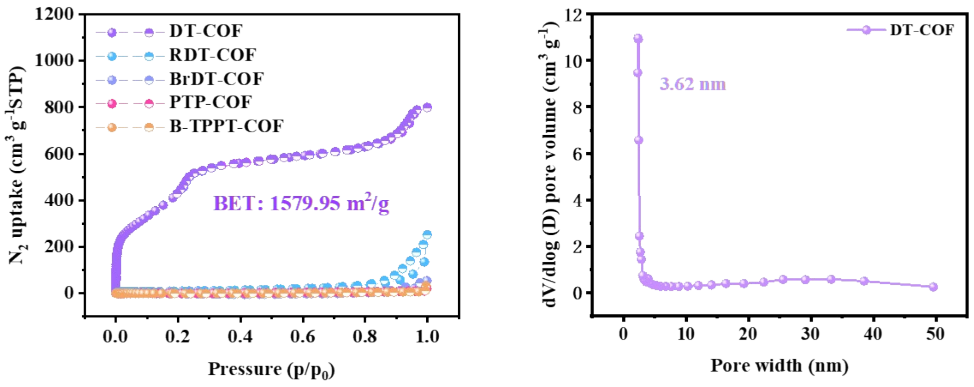

Figure S7 (a) N_2 sorption isotherms of DT-COF, RDT-COF, BrDT-COF, B-TPPT-COF, PTP-COF and PTP and (b)pore size distribution of DT-COF using Barrett-Joyner-Halenda (BJH) method.

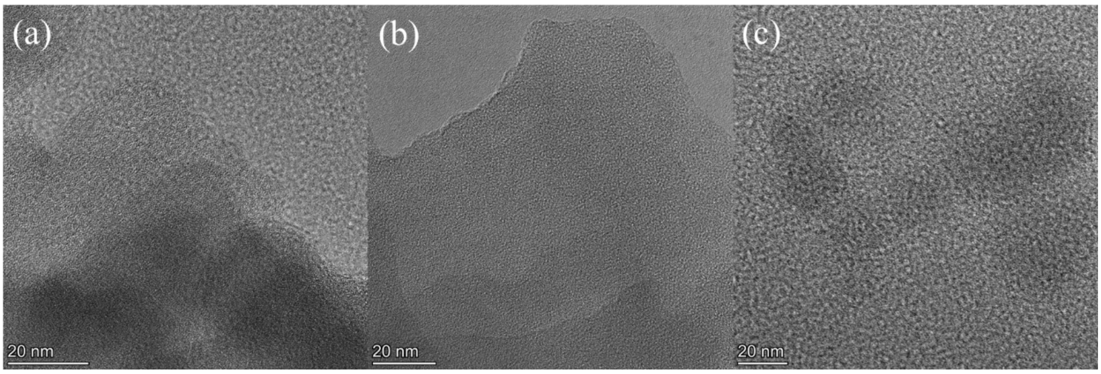

Figure S8 TEM images of (a) RDT-COF, (b) BrDT-COF and (c) PTP-COF.

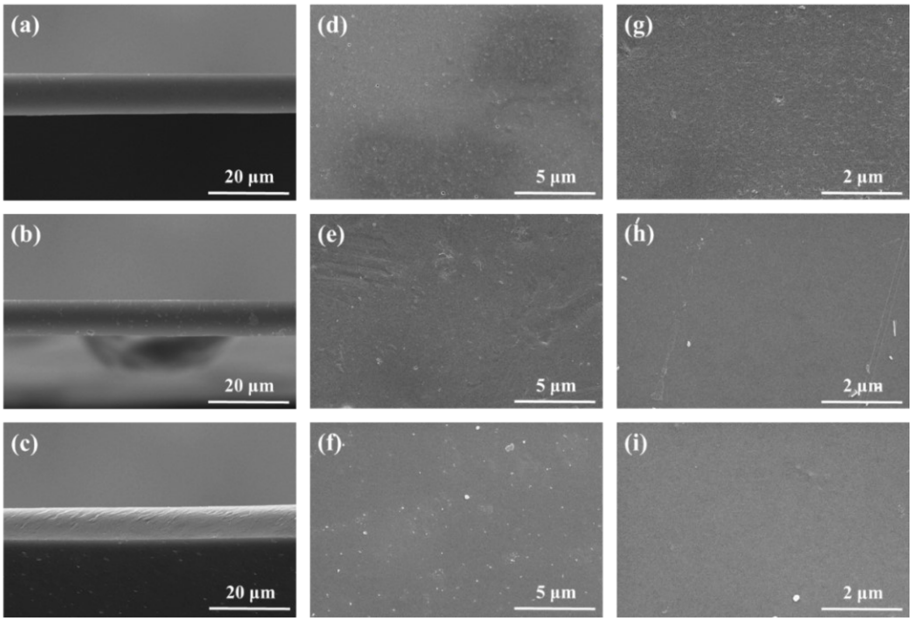

Figure S9 The SEM cross-section images of (a) B-TPPT-COF, (b) PTP-COF and (c) PTP and surface images of (d)-(g) B-TPPT-COF, (e)-(h) PTP-COF and (f)-(i) PTP.

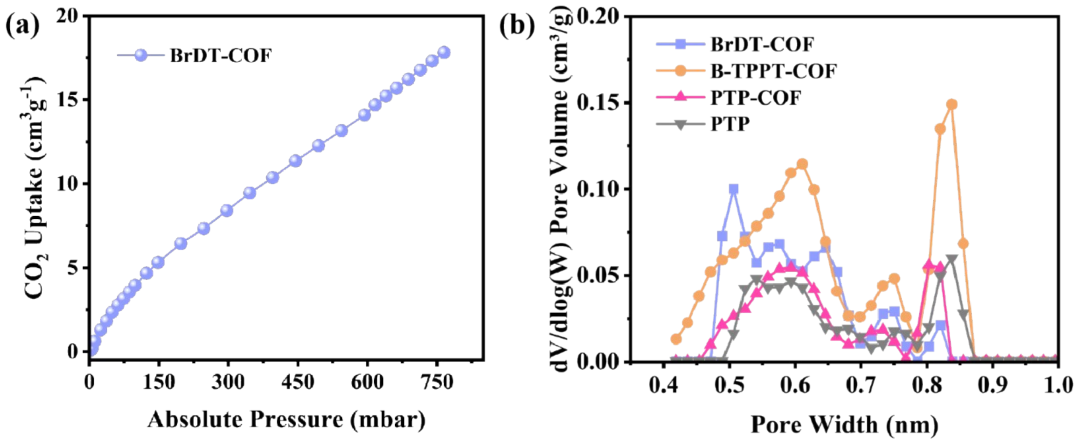

Figure S10 (a) CO_2 uptake of BrDT-COF, (b) the porosity distribution of PTP, PTP-COF, BrDT-COF and B-TPPT-COF based on density functional theory (DFT) calculations.

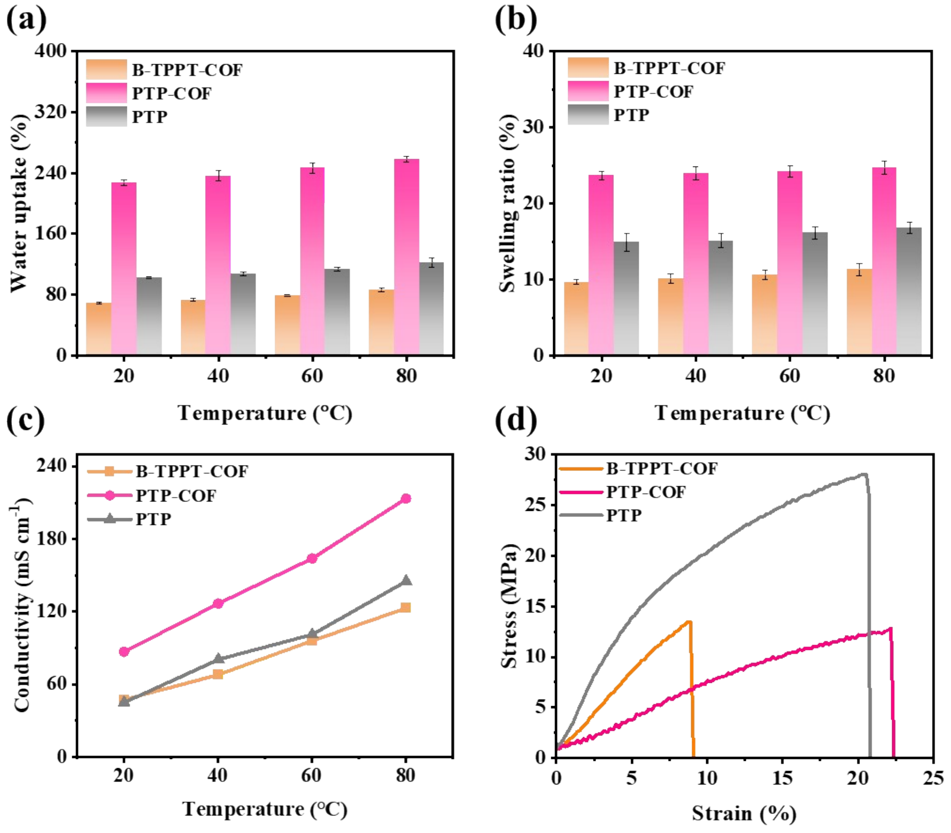

Figure S11 (a) water uptake, (b) swelling ratio, (c) hydroxide conductivity and (d) mechanical properties of B-TPPT-COF, PTP-COF and PTP membranes, respectively.

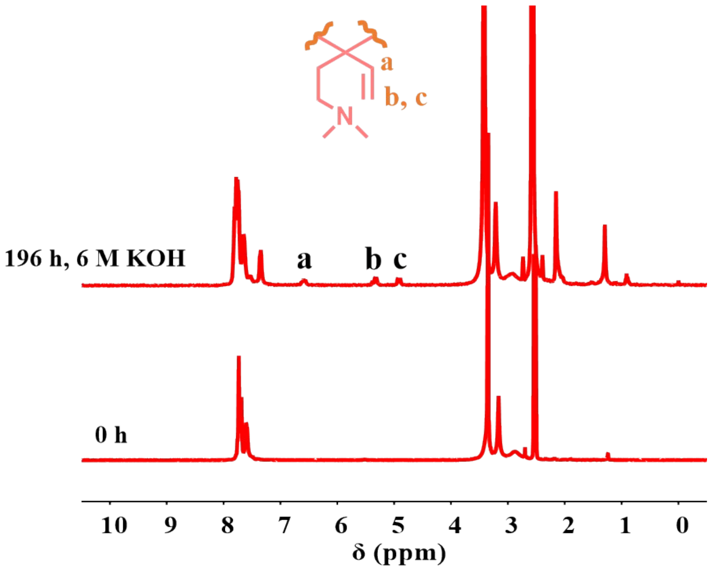

Figure S12 ^{1}H NMR spectra of B-TPPT-COF before and after the alkaline stability test.

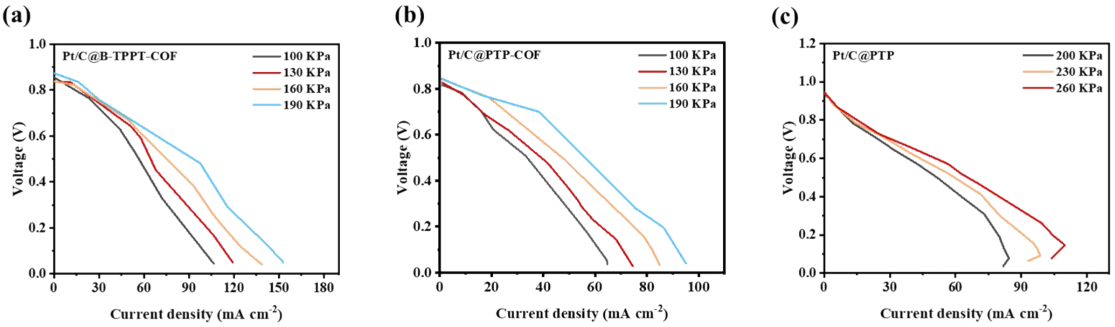

Figure S13 Polarization curves of (a) B-TPPT-COF electrode, (b) PTP-COF electrode and (c) PTP electrode at different pressure.

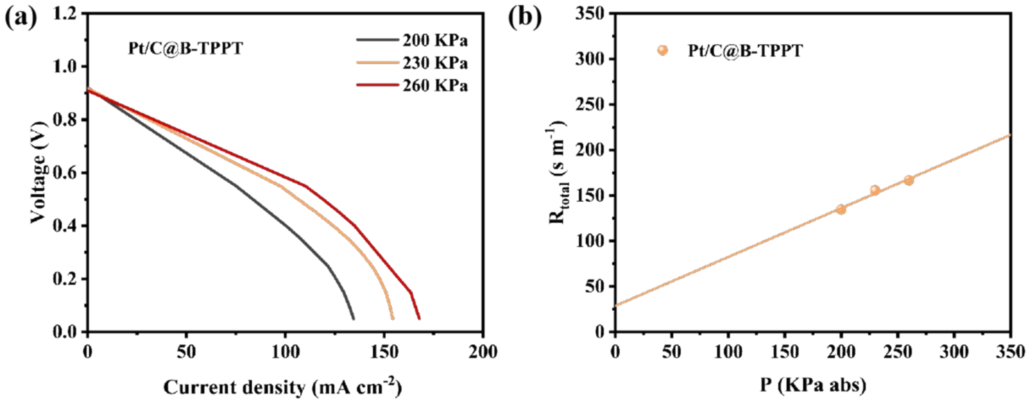

Figure S14 (a) Polarization curves of B-TPPT electrode at different pressure, (b) the total oxygen transport resistance of B-TPPT electrodes as a function of absolute gas pressure at 80 °C and 100% RH.
